# Clinical disease progression and biomarkers in Niemann–Pick disease type C: a prospective cohort study

**DOI:** 10.1186/s13023-020-01616-0

**Published:** 2020-11-23

**Authors:** Eugen Mengel, Bruno Bembi, Mireia del Toro, Federica Deodato, Matthias Gautschi, Stephanie Grunewald, Sabine Grønborg, Bénédicte Héron, Esther M. Maier, Agathe Roubertie, Saikat Santra, Anna Tylki-Szymanska, Simon Day, Tara Symonds, Stacie Hudgens, Marc C. Patterson, Christina Guldberg, Linda Ingemann, Nikolaj H. T. Petersen, Thomas Kirkegaard, Christine í Dali

**Affiliations:** 1 SphinCS GmbH, Institute of Clinical Science for LSD, Hochheim, Germany; 2 Regional Coordinator Centre for Rare Diseases, Academic Hospital Santa Maria Della Misericordia, Udine, Italy; 3 Vall D’Hebron University Hospital, Barcelona, Spain; 4 Ospedale Pediatrico Bambino Gesù, IRCCS, 00146 Rome, Italy; 5 Inselspital, University Hospital of Bern, Bern, Switzerland; 6 Metabolic Department, Great Ormond Street Hospital NHS Foundation Trust, Institute for Child Health, NIHR Biomedical Research Centre UCL, London, UK; 7 Centre for Inherited Metabolic Diseases, Copenhagen University Hospital (Rigshospitalet), Copenhagen, Denmark; 8 Reference Centre for Lysosomal Disease, Trousseau University Hospital, Paris, France; 9 Dr. Von Hauner Children’s Hospital, University of Munich, Munich, Germany; 10 Institute of Neurosciences, University Hospital of Montpellier, Montpellier, France; 11 Birmingham Children’s Hospital, Birmingham, UK; 12 Children’s Memorial Health Institute, Warsaw, Poland; 13 Clinical Trials Consulting & Training Limited, Buckingham, UK; 14 Clinical Outcomes Solutions Limited, Folkestone, UK; 15 Clinical Outcomes Solutions Inc, Tucson, AZ USA; 16 Mayo Clinic Children’s Center, Rochester, MN USA; 17 Orphazyme A/S, Copenhagen, Denmark

**Keywords:** Lysosomal storage disease, Niemann–Pick type C (NPC) disease, Observational study, NPC Clinical Severity Scale (NPCCSS), Heat shock protein, Reliability, Cholestane-triol, Biomarkers, Natural history of disease

## Abstract

**Background:**

Niemann–Pick disease type C (NPC) is a rare, progressive, neurodegenerative disease associated with neurovisceral manifestations resulting from lysosomal dysfunction and aberrant lipid accumulation. A multicentre, prospective observational study (Clinical Trials.gov ID: NCT02435030) of individuals with genetically confirmed NPC1 or NPC2 receiving routine clinical care was conducted, to prospectively characterize and measure NPC disease progression and to investigate potential NPC-related biomarkers versus healthy individuals. Progression was measured using the abbreviated 5-domain NPC Clinical Severity Scale (NPCCSS), 17-domain NPCCSS and NPC clinical database (NPC-cdb) score. Cholesterol esterification and heat shock protein 70 (HSP70) levels were assessed from peripheral blood mononuclear cells (PBMCs), cholestane-3β,5α-,6β-triol (cholestane-triol) from serum, and unesterified cholesterol from both PBMCs and skin biopsy samples. The inter- and intra-rater reliability of the 5-domain NPCCSS was assessed by 13 expert clinicians’ rating of four participants via video recordings, repeated after ≥ 3 weeks. Intraclass correlation coefficients (ICCs) were calculated.

**Results:**

Of the 36 individuals with NPC (2–18 years) enrolled, 31 (86.1%) completed the 6–14-month observation period; 30/36 (83.3%) were receiving miglustat as part of routine clinical care. A mean (± SD) increase in 5-domain NPCCSS scores of 1.4 (± 2.9) was observed, corresponding to an annualized progression rate of 1.5. On the 17-domain NPCCSS, a mean (± SD) progression of 2.7 (± 4.0) was reported. Compared with healthy individuals, the NPC population had significantly lower levels of cholesterol esterification (*p* < 0.0001), HSP70 (*p* < 0.0001) and skin unesterified cholesterol (*p* = 0.0006). Cholestane-triol levels were significantly higher in individuals with NPC versus healthy individuals (*p* = 0.008) and correlated with the 5-domain NPCCSS (Spearman’s correlation coefficient = 0.265, *p* = 0.0411). The 5-domain NPCCSS showed high ICC agreement in inter-rater reliability (ICC = 0.995) and intra-rater reliability (ICC = 0.937).

**Conclusions:**

Progression rates observed were consistent with other reports on disease progression in NPC. The 5-domain NPCCSS reliability study supports its use as an abbreviated alternative to the 17-domain NPCCSS that focuses on the most relevant domains of the disease. The data support the use of cholestane-triol as a disease monitoring biomarker and the novel methods of measuring unesterified cholesterol could be applicable to support NPC diagnosis. Levels of HSP70 in individuals with NPC were significantly decreased compared with healthy individuals.

**Trial registration:**

CT-ORZY-NPC-001: ClincalTrials.gov NCT02435030, Registered 6 May 2015, https://clinicaltrials.gov/ct2/show/NCT02435030; EudraCT 2014–005,194-37, Registered 28 April 2015, https://www.clinicaltrialsregister.eu/ctr-search/trial/2014-005194-37/DE. OR-REL-NPC-01: Unregistered.

## Background

Niemann–Pick disease type C (NPC) is a rare, progressive, neurodegenerative disease caused by autosomal recessive mutations in either the *NPC1* (~ 95% of cases) or the *NPC2* (~ 5% of cases) gene [1, 2]. The gene products, NPC1 and NPC2, are lysosomal/endosomal proteins, responsible for intracellular lipid transport and homeostasis [1, 3–5]. NPC is a disease characterized by lysosomal dysfunction, which leads to accumulation of lipids such as unesterified cholesterol and sphingolipids in the late lysosomal/endosomal compartments within multiple organs, predominantly the liver, spleen, lungs and brain [1, 6].

Overall, the clinical presentation of NPC varies widely and depends on age of onset of neurological symptoms [1, 7, 8]. Individuals with early infantile onset NPC frequently have isolated hepato-splenomegaly and cholestatic jaundice, whereas individuals with a later onset are more likely to have moderate or subclinical organomegaly [1]. As NPC disease advances, progressive and severe neurological symptoms develop that affect gross motor skills, swallowing ability cognitive functions [7], and life expectancy is shortened [1, 7, 9–11]. Phenotypes are categorized by the age of onset and the progression rate of neurological symptoms: the severe and lethal early–infantile and late–infantile phenotypes are most often associated with the presence of functional null mutations, in which loss-of-function, frameshift, splicing or premature stop mutations on both alleles of *NPC1*/*NPC2* result in a non-functional translation product [12]. Individuals with juvenile and adult NPC phenotypes generally carry missense mutations on at least one allele [12].

In rare diseases, real-world data on the features and natural progression of a disease and the frequency of associated adverse events (AEs) and comorbidities are important to understand and information from non-therapeutic and observational studies can therefore help guide the design of clinical trials [13]*.* This is also the case in NPC, where a better understanding of natural history and genotypic and phenotypic variability might aid in the design of clinical trials.

**Fig. 1:**
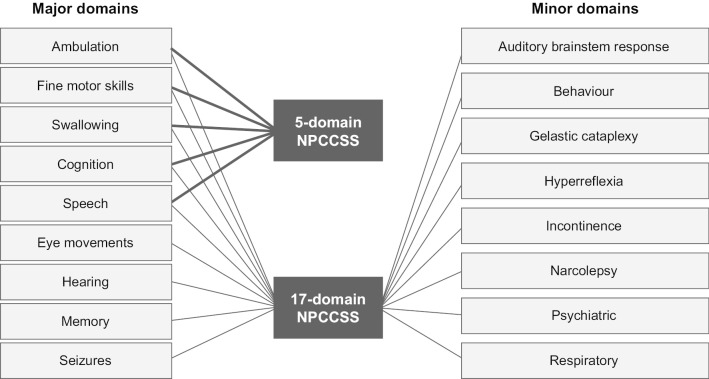
Sub-domains of the 5-domain and 17-domain Niemann–Pick type C Clinical Severity Scale. These are the five domains selected by individuals with NPC, their caregivers and NPC experts as the most clinically relevant domains. NPC: Niemann–Pick disease type C; NPCCSS: NPC Clinical Severity Scale

A clinical staging system for NPC was first proposed in 1992 based on a small group of intensely studied patients but was never widely adopted [14]. In 2006, a 4-domain scale was developed from the analysis of a cohort of 30 individuals with NPC; subsequent scales have built on this original work, with varying modifications [15]. The 17-domain NPC Clinical Severity Scale (NPCCSS), a measure of NPC disease severity and progression, was introduced in 2010, based on a cohort of 18 then-current individuals with NPC and 19 historical cases from a study by the National Institutes of Health [16]. Although this scale has been used in several clinical trials, notably Ory et al. [17], it can be difficult to implement in everyday clinical practice owing to its complexity. In addition, several of the domains included in this instrument—seizures, gelastic cataplexy and psychiatric manifestations—may be influenced by symptomatic treatment and could potentially confound studies of disease-modifying agents. To reduce variability and increase the suitability for use in clinical trials, an abbreviated version of the 17-domain NPCCSS was developed comprising five domains (the 5-domain NPCCSS) selected by individuals with NPC, their caregivers and NPC experts as the most clinically relevant domains [18]. These five domains are ambulation, cognition, fine motor skills, speech and swallowing (Fig. 1). Although a high correlation (Spearman’s correlation coefficient = 0.93) has previously been found between the 5-domain NPCCSS and the 17-domain NPCCSS [18], there is a need to provide better support for the performance of this revised tool in assessing NPC disease severity and progression. Therefore, as part of the validation process, to assess the reliability of the 5-domain NPCCSS as a useful measure and endpoint in clinical studies, a study was performed to determine the accuracy of the measurements between raters (inter-rater reliability) and within raters (intra-rater reliability over time) [19, 20].

In addition to the development of tools to measure NPC disease progression, diagnostic tests of NPC have also evolved over time [21]. Lysosomal lipid accumulation has traditionally been used for diagnostic purposes for decades, using analysis of unesterified cholesterol by filipin staining of cultured patient fibroblasts as a static marker of lipid storage burden [1, 21]. Traditionally, filipin staining, which highlights the accumulation of unesterified cholesterol, requires cultured patient fibroblasts and expert interpreters [21]. Although filipin staining is an invasive procedure that is laborious and time-consuming, with poor specificity and sensitivity, it remains the gold standard method of diagnosis [21], especially when genetic testing yields variants of unknown significance. The increase in unesterified cholesterol in the late endosome/lysosome further leads to the generation of oxidated cholesterol derivatives in the serum of individuals with NPC. Some of these oxidated cholesterol derivatives, like cholestane-3β,5α,6β-triol (cholestane-triol), have been demonstrated to aid diagnosis of NPC and cholestane-triol levels correlate with NPC disease severity and age of NPC disease onset [22]. Defects in the transport of unesterified cholesterol to the endoplasmic reticulum for esterification reduces cholesterol esterification in skin fibroblasts of individuals with NPC compared with healthy individuals. Cholesterol esterification has been proposed to reflect NPC protein function and might therefore be used as a diagnostic biomarker for NPC [23–25].

In contrast to the established biomarkers of NPC, less is currently understood about the role of the heat shock response (HSR) in individuals with NPC. The HSR, regulated by the heat shock factor 1 (HSF1) transcription factor, is part of a natural cellular defence mechanism against stress-induced protein misfolding, protein homeostasis disruption and lysosomal dysfunction [9, 26]. HSP70, the most highly expressed heat shock protein (HSP) under cellular stress, promotes protein refolding and lysosomal function through its interactions with the lysosomal sphingolipid metabolic pathways [9, 27–34]. Specifically, HSP70 has been shown to aid the maturation of both wild type and missense mutated NPC1 [28]. An insufficient HSR has been associated with chronic neurological diseases that involve the accumulation of misfolded proteins and disturbed cellular homeostasis [26, 35–37]. HSP70 and active HSF1 levels are reduced in the brain of *Npc1*^*−/−*^ mice, potentially linking the HSR to aberrant lysosomal function [27]. Correlations between improved Purkinje cell survival rates and the presence of HSPs in central and nodular cerebellar zones have also long been recognized in mice [28, 38]. The HSR and HSP70, in particular, represent significant potential as a therapeutic target for NPC; therefore, the characterization of HSP70 expression levels in individuals with NPC during the study was of interest.

### Aims

To understand disease progression patterns in individuals with NPC based on the 5-domain NPCCSS, a prospective, observational study was conducted. The study also aimed to characterize novel biomarkers associated with the altered NPC1/2 protein function and lipid metabolism in NPC disease, monitoring their expression longitudinally, in order to establish a set of biomarkers for NPC and for interventions targeting the HSP system in NPC (Orphazyme protocol number: CT-ORZY-NPC-001; ClinicalTrials.gov ID: NCT02435030) [39].

Additionally, to assess inter- and intra-rater reliability of the 5-domain NPCCSS as a measure of disease severity in individuals with NPC, the agreement between 5-domain NPCCSS total scores and individual items was evaluated (Orphazyme protocol number: OR-REL-NPC-01).

## Results

As described in the Methods, two studies were conducted: the prospective, multicentre observational study (CT-ORZY-NPC-001) to measure disease progression on the 5-domain NPCCSS and NPC disease biomarkers; and the reliability study to assess the inter- and intra-rater reliability of the 5-domain NPCCSS score (OR-REL-NPC-01). The results of these studies are presented below.

### Observational study (CT-ORZY-NPC-001)

#### Study participants

**Fig. 2:**
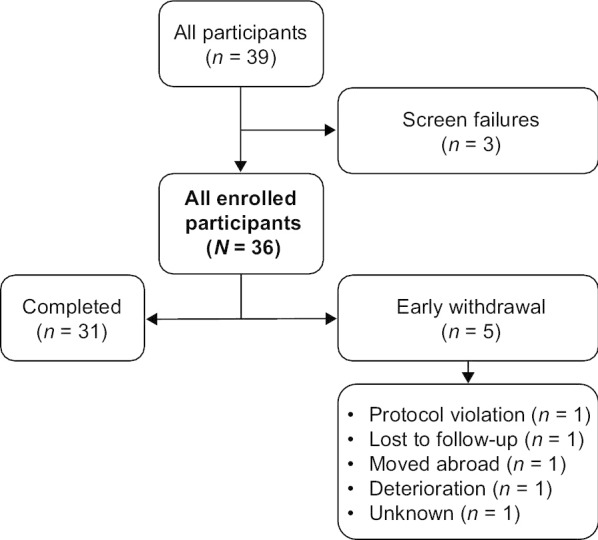
Participant flow diagram. *N* = overall number of enrolled participants; *n* = number of participants per category. One participant who withdrew early had an end-of-trial visit and is included in efficacy assessments

Thirty-six individuals were enrolled in the study across 12 clinical sites in seven countries; 31 individuals completed the study (Fig. 2) over the observation period of 6–14 months. All individuals had confirmed mutations of the *NPC1* gene. There were five withdrawals from the study for the following reasons: one protocol violation; one lost to follow-up; one international relocation; one discontinuation due to NPC deterioration; and one early withdrawal for unknown reasons. The participant who withdrew early owing to the international relocation still had an end-of-trial visit and provided data for the assessments (Fig. 2). The mean (± standard deviation [SD]) length of follow-up time for all individuals was 10.2 months (± 3.1).

The mean age of the population was 9.9 years (SD ± 4.6); individuals were aged 2–18 years; the majority of the population was Caucasian (33/36; 91.7%) and 58.3% (21/36) were female. With regard to phenotype characterization, 16.7% (6/36) presented with early–infantile onset (< 2 years old), 52.8% (19/36) presented with late–infantile onset (2– < 6 years old) and 30.6% (11/36) presented with juvenile onset (6– < 15 years old). The mean (± SD) time elapsed since first NPC symptom was 6.7 years (± 3.7) and the mean time elapsed since diagnosis was 5.3 years (± 3.4) (Table 1).

**Table 1:** Participant demographics and disease characteristics (all enrolled participants)

Demographic data		All individuals (*N* = 36)
Age, years	Mean (SD)	9.9 (4.6)
	Median	9.5
	Range	2.0–18.0
Sex, *n* (%)	Male	15 (41.7%)
	Female	21 (58.3%)
Ethnicity, *n* (%)	Caucasian	33 (91.7%)
	Asian	1 (2.8%)
	Other	2 (5.6%)
Height, cm	Mean (SD)	134.1 (24.9)
	Median	136.0
	Range	82.0–176.0
Height-for-age Z score	Mean (SD)	− 0.14 (1.36)
	Median	0.06
	Range	(− 3.38–2.67)
Weight, kg	Mean (SD)	34.63 (15.57)
	Median	32.35
	Range	10.00–71.70
Body mass index, kg/m^2^	Mean (SD)	18.0 (2.6)
	Median	18.0
	Range	14.1–23.1
Age at first NPC symptom, years	Mean (SD)	3.8 (3.4)
	Median	3.0
	Range	0.0–14.0
Phenotype characterization, *n* (%)	Early–infantile onset (< 2 years old)	6 (16.7%)
	Late–infantile onset (2– < 6 years old)	19 (52.8%)
	Juvenile onset (6– < 15 years old)	11 (30.6%)
Time since first NPC symptom, years	Mean (SD)	6.7 (3.7)
	Median	6.4
	Range	1.7–15.8
Time since NPC diagnosis, years	Mean (SD)	5.3 (4.0)
	Median	3.5
	Range	0.2–15.8
Currently treated with miglustat, n (%)	Yes	30 (83.3%)
	No	6 (16.7%)
Historic NPC-cdb score	Mean (SD)	40 (21)
	Median	38
	Range	4–91

NPC: Niemann–Pick disease type C; NPC-cdb: NPC clinical database; SD: standard deviation

Regarding baseline disease characteristics, the most commonly reported neurological symptoms, provided by referring clinicians as current medical conditions, was ataxic clumsiness/impaired coordination (31/36; 86.1%), and vertical supranuclear gaze palsy (29/36; 80.6%). In the clinician assessments, the specific term of ataxia was only listed for 2 of 36 individuals (5.6%); however, when assessed using the NPC-cdb tool, ataxia was found to be present in 28 individuals (77.8%). Additionally, comorbid neurological disorders were reported in 11 of the 36 individuals (30.6%) under the preferred terms of epilepsy (5/36; 13.9%), cataplexy (2/36; 5.6%), seizure (2/36; 5.6%), hypotonia (1/36; 2.8%) and resting tremor (1/36; 2.8%). Miglustat was being administered as part of routine clinical care in 30 of the 36 individuals (83.3%), and 9 individuals (25.0%) were receiving concomitant anti-epileptic medication (lamotrigine, levetiracetam and/or topiramate).

#### Clinical progression

**Fig. 3:**
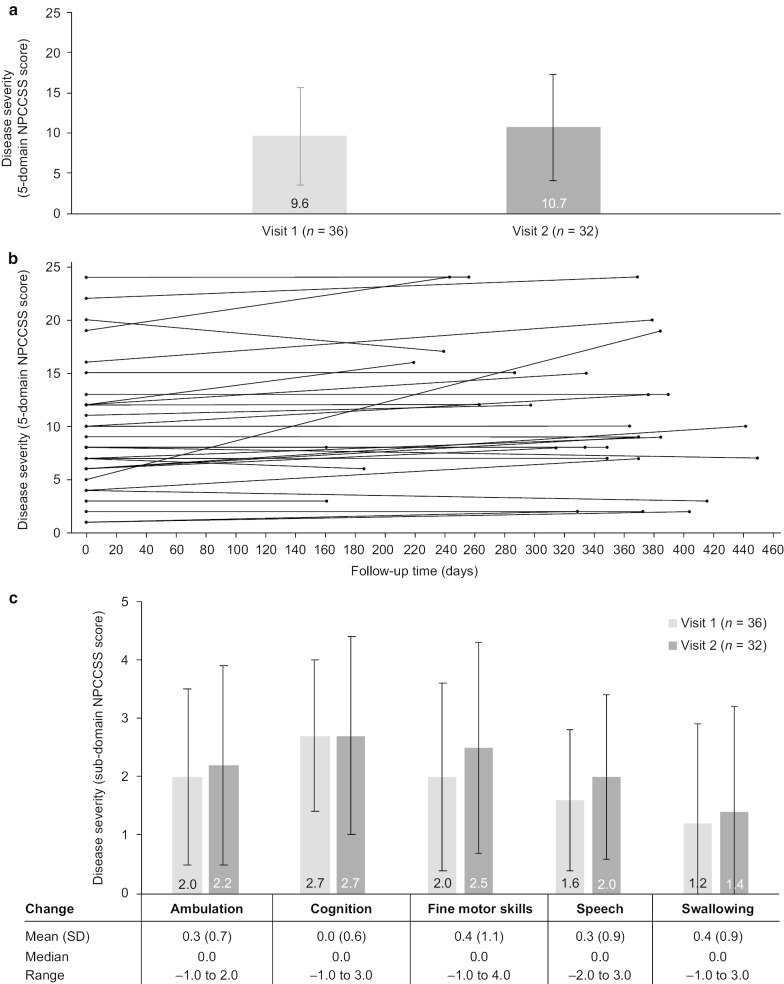
5-domain NPCCSS scores at Visit 1 and Visit 2. **a** Mean (SD) overall scores at Visit 1 and Visit 2 for all participants.* **b** Change in overall scores from Visit 1 to Visit 2 by length of follow-up time. **c** Mean (SD) individual scores for the five key sub-domains at Visit 1 and Visit 2, with mean change. NPCCSS: Niemann–Pick disease type C Clinical Severity Scale; SD: standard deviation. *One participant who withdrew early had relocated, but had an end-of-trial visit and is included in efficacy assessments. According to the statistical analysis plan, the NPC-001 study ended at a study site once the interventional NPC-002 study commenced at that specific site. The NPC-002 study had not started at the site of this particular participant when they relocated, therefore the end of study visit was planned (prior to completion of the study). The participant had a planned withdrawal visit after 162 days. Of the study withdrawals, only this participant had an end of study visit. Their screening visit and end of study visit are therefore included as per statistical analysis plan

At Visit 1, the mean (± SD) baseline disease severity scores were 9.6 (± 6.0) for the 5-domain NPCCSS and 16.7 (± 9.6) for the 17-domain NPCCSS. The mean (± SD) increase in the total 5-domain NPCCSS was 1.4 (± 2.9), representing a mean (± SD) change per 6 months of 0.75 (± 1.58; annualized progression = 1.5 points) (Fig. 3a). Except for cognition, each of the individual five domains showed an increase in score from Visit 1 to Visit 2 (Fig. 3b). For the 17-domain NPCCSS, there was a mean (± SD) increase of 2.7 (± 4.0), representing a mean (± SD) change per 6 months of 1.47 (± 2.25).

Disease progression was also evidenced using the NPC clinical database (NPC-cdb) score, for which the baseline score (± SD) was 39.4 (± 20.0) and there was a mean (± SD) increase of 5.0 (± 7.9). Using the Pareto principle for evaluation of the EuroQol 5-Dimension 3-Level Youth Proxy version questionnaire (EQ-5D-3L Y) assessment tool, of the 30 individuals who completed repeat assessment, 12 individuals (40.0%) had a worse score at Visit 2 compared with Visit 1 and 7 (23.3%) each had a better score or an equal score at Visit 2 compared with Visit 1 (Table 2).

**Table 2:** Pareto principle classifications for evaluation of the EQ-5D-3L Y from Visit 1 to Visit 2

Classification	Number of individuals (*n* = 30)
Better (at least one dimension has improved, and no worsening is seen in any other dimension), *n* (%)	7 (23.3%)
Worse (at least one dimension has worsened, and no improvements are seen in any other dimension), *n* (%)	12 (40.0%)
Same (the health profiles are the same; there has been no change in health state), *n* (%)	7 (23.3%)
Mixed (the dimensions are better on one dimension, but worse on another), *n* (%)	4 (13.3%)

EQ-5D-3L Y: EuroQol 5-Dimension 3-Level Youth Proxy version questionnaire

#### Genotype analysis

Based on historical genotype examinations of the ClinVar and NPC-db2 databases [40], there were 34 distinct previously reported alleles and 8 distinct unreported mutation alleles of the *NPC1* gene, within the study population of 36 enrolled participants. Most arose from substitution mutations at the DNA level (87.5%; n = 63 alleles), which in most cases resulted in missense mutations at the protein level (76.4%; n = 55 alleles) (Table 3). For two mutations, the change on the protein level could not be determined. Functional null mutations, such as frameshift, splicing or premature stop mutations, were present in 20.8% (n = 15) of alleles; one individual had a double functional null mutation: A1108fs/A1108fs. This individual was 2.5 years old at enrolment and exhibited a severe disease progression of 14 points during an observation period of 13 months.

**Table 3:** Summary of genotypic data from 72 alleles in 36 enrolled individuals

	NPC alleles (*n* = 72)
Total number of different alleles	42
Known mutations	34
New mutations	8
DNA level mutations	
Deletion	4 (5.6%)
Insertion	5 (6.9%)
Substitution	63 (87.5%)
Protein level mutations	
Frameshift	9 (12.5%)
Missense	55 (76.4%)
Splice	3 (4.2%)
Stop codon	3 (4.2%)
Unknown	2 (2.8%)

NPC: Niemann–Pick disease type C

#### Biomarkers

**Fig. 4:**
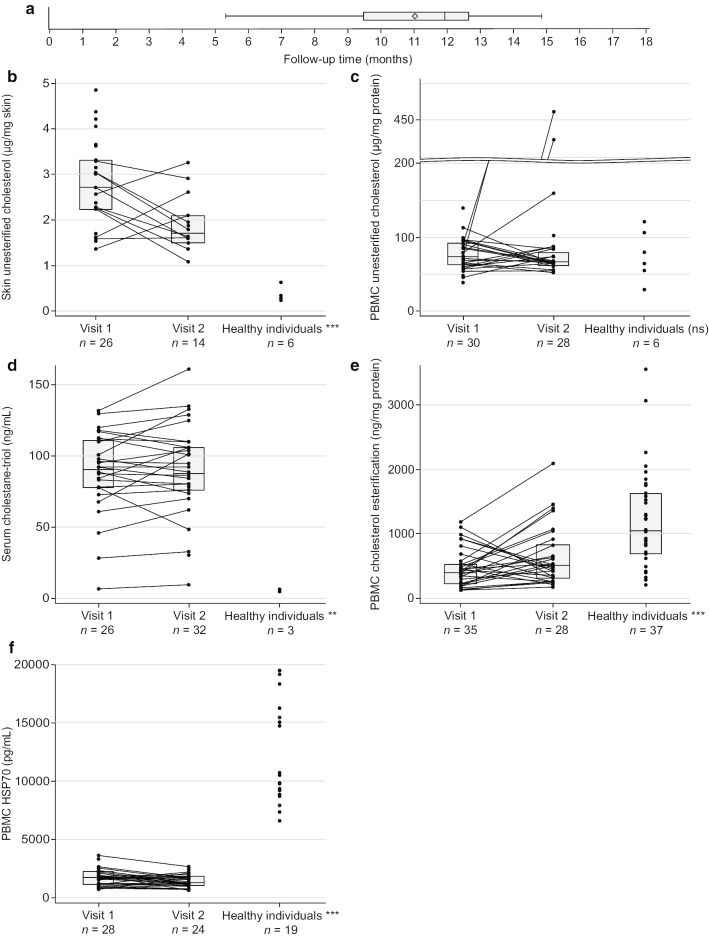
Biomarker results at Visit 1 and Visit 2 in comparison to healthy individuals. **a** Median and interquartile lengths of average follow-up time. **b** Skin unesterified cholesterol, *p* = 0.0006. **c** PBMC unesterified cholesterol, *p* = 0.8979. **d** Serum cholestane-triol, *p* = 0.008. **e** PBMC cholesterol esterification, *p* < 0.0001. **f** PBMC HSP70, *p* < 0.0001. HSP70: heat shock protein 70; NPCCSS: Niemann–Pick disease type C Clinical Severity Scale; NS: not significant; PBMC: peripheral blood mononuclear cell

Values at Visits 1 and 2, changes in parameters per 6 months, and values for healthy individuals for all biomarkers are presented in Table 4 and Fig. 4.

**Table 4:** Change in disease biomarkers over the 6–14-month observation period compared with those of healthy individuals

	Skin unesterified cholesterol (μg/mg skin)	PBMC unesterified cholesterol (μg/mg protein)	Serum cholestane-triol (ng/mL)	PBMC cholesterol esterification (ng/mg protein)	PBMC HSP70 (pg/mL)
Visit 1, *n*	26	30	28	35	28
Mean (± SD)	2.79 (± 0.96)	77.56 (± 21.53)	88.31 (± 28.66)	462.14 (± 283.60)	1804.09 (± 713.67)
Visit 2, *n*	14	28	32	28	24
Mean (± SD)	1.89 (± 0.64)	98.45 (± 99.56)	88.52 (± 31.56)	616.89 (± 443.10)	1469.94 (± 581.21)
*p* value for the comparison between visits*	0.1748	0.8015	0.3069	0.1212	0.0121
Change in parameter per 6 months, *n*	11	23	26	27	20
Mean (± SD)	–0.23 (± 0.47)	25.94 (± 97.56)	2.64 (± 10.69)	57.41 (± 303.81)	–225.36 (± 411.76)
Healthy individuals, *n*	6	6	3	37	19
Mean (± SD)	0.36 (± 0.14)	76.39 (± 33.82)	5.97 (± 1.13)	1197.15 (± 763.65)	12,310.21 (± 4247.23)
*p* values compared with healthy individuals*	0.0006	0.8979	0.008	< 0.0001	< 0.0001

^*^Comparisons between visits utilized the Wilcoxon–Mann–Whitney test. Comparisons between individuals with NPC and healthy individuals utilized the Wilcoxon signed-rank test

HSP70: heat shock protein 70; PBMC: peripheral blood mononuclear cell; SD: standard deviation

**Fig. 5:**
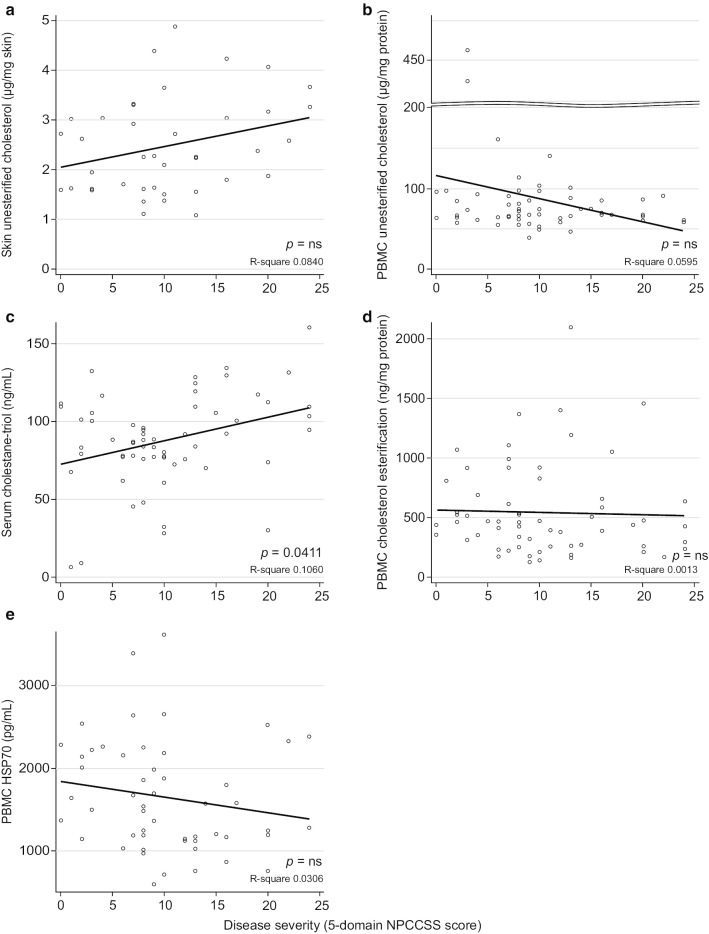
Biomarker correlations with disease severity as measured by the 5-domain NPCCSS. **a** PBMC unesterified cholesterol. **b** Skin unesterified cholesterol. **c** Serum cholestane-triol. **d** PBMC cholesterol esterification. **e** PBMC HSP70. HSP70: heat shock protein 70; NPCCSS: Niemann–Pick disease type C Clinical Severity Scale; PBMC: peripheral blood mononuclear cell

Unesterified cholesterol levels in the skin biopsy samples were numerically decreased from Visit 1 to Visit 2 (*p* = 0.1748), with a mean (± SD) change in parameter per 6 months of –0.23 μg/mg skin (± 0.47). Moreover, equivalent measurements in a cohort of healthy individuals (*n* = 6) of 0.36 μg/mg skin (± 0.14) were significantly lower compared with individuals with NPC (*p* = 0.0006; Table 4, Fig. 4b). This result demonstrates that unesterified cholesterol measured by LC–MS/MS in skin can be successfully used as a novel method to measure cholesterol burden. In PBMCs, unesterified cholesterol levels were numerically increased between Visits 1 and 2 (*p* = 0.8015) and showed a mean (± SD) change in parameter per 6 months of 25.94 μg/mg protein (± 97.56); equivalent measurements in a cohort of healthy individuals (*n* = 6) of 76.39 ng/mg protein (± 33.82) were also numerically lower compared with individuals with NPC (Table 4, Fig. 4c). However, there was considerable variability in the study population owing to the presence of two outliers distorting the summary statistics. There were no significant correlations found between disease severity as measured by the 5-domain NPCCSS and either skin or PBMC unesterified cholesterol (Fig. 5a, b).

Mean (± SD) serum cholestane-triol levels in individuals with NPC at both Visits 1 and 2 (88.31 ng/mL [± 28.66] and 88.52 [± 31.56], respectively) were significantly higher than the mean (± SD) serum cholestane-triol levels of 5.97 ng/mL (± 1.13) observed in a cohort of healthy individuals (*n* = 3; *p* = 0.008). There was also a numerical increase between Visits 1 and 2 (*p* = 0.3069), with a mean (± SD) change per 6 months of 2.64 ng/mL (± 10.69). These findings are consistent with those previously published elsewhere in the literature [22, 41] (Table 4, Fig. 4d). Furthermore, cholestane-triol levels correlate (Spearman’s correlation coefficient = 0.265, *p* = 0.0411) with 5-domain NPCCSS scores (Fig. 5c).

Cholesterol esterification levels in PBMC, a measure of the NPC protein functionality, were numerically increased from Visit 1 to Visit 2 (*p* = 0.1212), and showed a mean (± SD) change per 6 months of 57.41 ng/mg protein (± 303.81). This analytical method was originally described by Vanier in 1988 and has been used in support of NPC diagnosis [25]. Cholesterol esterification levels in samples from those with NPC were significantly lower than those from a cohort of healthy individuals (n = 37; *p* < 0.0001), reflecting the reduced NPC protein function in individuals with NPC (Table 4, Fig. 4e). There were no significant correlations found between disease severity and cholesterol esterification (Fig. 5d).

Across the study population, mean (± SD) HSP70 expression levels changed from Visit 1 (1804.09 pg/mL [± 713.67]) to Visit 2 (1469.94 pg/mL [± 581.21]; *p* = 0.0121), and had a mean (± SD) change per 6 months of –225.36 pg/mL (± 411.76). In all but one individual, HSP70 expression levels were significantly decreased in PBMCs of the individuals with NPC compared with those observed in a cohort of healthy individuals (*n* = 19; *p* < 0.0001) (Table 4, Fig. 4f). There were no significant correlations found between disease severity and HSP70 (Fig. 5e).

#### Adverse events

There were 120 AEs occurring in 25 of 36 individuals (61.1%); most of these AEs were mild to moderate in severity. The majority of AEs were classed as infections (18/36 individuals; 50.0%), with the most common being rhinitis (8/36, 22.2%). Epilepsy accounted for two AEs in one of 36 individuals (2.8%). There were five serious adverse events (SAEs) that occurred in three individuals; all of these SAEs were infections. No AEs or SAEs resulted in death or discontinuations (Table 5).

**Table 5:** Summary of adverse events and serious adverse events (all enrolled participants)

	Number of individuals (*N* = 36)
Adverse events	-
Total	25 (69.4%)
Mild	23 (63.9%)
Moderate	15 (41.7%)
Severe	5 (13.9%)
Most common adverse events experienced by > 5% of participants	-
Rhinitis	8 (22.2%)
Diarrhoea	7 (19.4%)
Pyrexia	6 (16.7%)
Nasopharyngitis	5 (13.9%)
Bronchitis	3 (8.3%)
Gastroenteritis	2 (5.6%)
Cough	2 (5.6%)
Vomiting	2 (5.6%)
Hepatomegaly	2 (5.6%)
Pharyngitis	2 (5.6%)
Skin infection	2 (5.6%)
Pain in extremities	2 (5.6%)
Headache	2 (5.6%)
Serious adverse events	
Total	3 (8.3%)
Wound infection	2 (5.6%)
Streptococcus infection	1 (2.8%)
Scarlet fever	1 (2.8%)
EBV infection	1 (2.8%)
Discontinuations owing to adverse events	0 (0.0%)

EBV: Epstein–Barr virus

### Reliability study of the 5-domain NPCCSS (OR-REL-NPC-01)

A total of four video recordings of individuals with NPC were used in the reliability study that was part of the validation process of the 5-domain NPCCSS. The videos represented a wide range of disease severities 5-domain NPCCSS scores (mean values ranged from 4.46 to 18.85; Table 6) and ages (8, 10, 12 and 17 years). To assess inter- and intra-rater reliability of the 5-domain NPCCSS, a group of 13 NPC expert clinicians provided initial and repeat ratings of the four videos, with at least a 3-week interval between repeat ratings. The resulting intraclass correlation coefficients (ICCs) were high between the initial and repeat time points (intra-rater reliability ICC = 0.937), as well as between clinicians (ICC = 0.995). For the individual domains, the coefficients were above 0.70 for all domains at the initial time point (range 0.763–0.954). At the repeat time point, ambulation fell slightly below the criterion threshold (coefficient of concordance = 0.681), with all other domains above the criterion threshold (range 0.723–0.949) (Table 6).

**Table 6:** Item-level coefficient of concordance for the 5-domain NPCCSS

Domain	Kappa coefficient of concordance	
	Initial rating	Repeat rating
Ambulation	0.785	0.681
Cognition	0.917	0.883
Fine motor skills	0.763	0.723
Speech	0.954	0.949
Swallowing	0.813	0.858

Kappa coefficients of concordance for initial and repeat ratings for each domain

NPCCSS: Niemann–Pick type C disease Clinical Severity Scale

As a sensitivity analysis, an evaluation of the clinician ratings using item-response theory analyses was conducted using participant videos and 5-domain NPCCSS scores, which indicated strong agreement across the clinician group (57.6% exact agreement; 0.87 correlation across ratings; sample error = 0.04). More specifically, this evaluation used the Many-Facets item analysis, an extension of Rasch measurement models for rater-mediated data [42]. In terms of the fit of the participant videos, all videos fit the model, none were misfitting (infit value ≥ 1.5; range 0.76–1.11) and the correlation across ratings was 0.73 with an error rate of 0.02. For the individual domains, there were no misfitting domains (infit value ≥ 1.5; range 0.72–1.16) and the correlation across ratings was 0.85 with an error rate of 0.01.

Additionally, in the rating scale analysis, conducted using the same Many-Facets model, monotonic increases in severity rating from lowest (verbal response option = 0; average person measure = − 4.59) to most severe (verbal response option = 5; average person measure = 3.53) were observed. Agreement between the participant severity ratings was observed between the classical descriptive statistics (ICC) on the 5-domain NPCCSS and the item-level Many-Facets analysis. Facets measures were as follows: participant C = 3.30 (most severe); participant B = 0.22; participant A = − 1.32; participant D = − 2.20 (least severe) (Table 7).

**Table 7:** Mean 5-domain NPCCSS score by participant videos

Participants*	MEAN 5-domain NPCCSS score (± SD)	
	Initial rating	Repeat rating
Participant A	5.85 (2.115)	7.00 (1.633)
Participant B	11.08 (2.326)	10.46 (1.713)
Participant C	18.15 (1.772)	18.85 (1.068)
Participant D	4.46 (1.391)	4.77 (2.204)
*p* value	< 0.0001	< 0.0001

* Facets measures were as follows: participant C = 3.30 (most severe); participant B = 0.22; participant A = –1.32; participant D = –2.20 (least severe)

NPCCSS: Niemann–Pick type C disease Clinical Severity Scale

## Discussion

In the field of rare diseases, prospective observational studies are vital for establishing patterns of disease progression and to help inform and shape relevant outcome measures for future clinical trials [43]. In this prospective, observational cohort study of NPC, the disease severity of the study population worsened from Visit 1 to Visit 2; this applies to all the clinical scores assessed (5-domain NPCCSS, 17-domain NPCCSS and NPC-cdb). The mean (± SD) change on the 5-domain NPCCSS at 6 months was 0.75 (± 1.58), corresponding to an annualized disease progression of 1.5 within the cohort. The mean (± SD) change on the 17-domain NPCCSS at 6 months and the mean (± SD) overall change on the NPC-cdb were 1.47 (± 2.25) and 5.0 (± 7.9), respectively. The findings from the NPC-001 study therefore support the characterization of NPC as a heterogenous, progressive neurodegenerative disease.

The use of the 5-domain NPCCSS has previously been supported by Cortina-Borja et al*.* 2018 [18]. The study reported that these five domains (ambulation, cognition, fine motor skills, speech and swallowing) were determined by individuals with NPC, caregivers and clinicians, to be the most clinically relevant and were highly correlated with the total 17-domain NPCCSS (Spearman’s correlation coefficient = 93%) [18]. The findings from the Cortina-Borja et al*.* cohort indicate that the 5-domain NPCCSS reflects disease progression measured by the 17-domain NPCCSS; further work is underway to validate and to confirm the patients and clinicians view on clinical meaningful changes on the 5-domain NPCCSS and is documented in the NPC Patient-Focused Drug Development report to the Food and Drug Administration [44].

Disease progression observed on the 17-domain NPCCSS was consistent with other NPC populations. In the population studied by Ory et al*.* [17], the change in disease progression per 12 months on the overall 17-domain NPCCSS was 2.92 (standard error of the mean [SEM] = 0.27), which is consistent with the mean (± SD) reported progression rate per 6 months in this current study of 1.47 (± 2.25) of a heterogeneous study population. As mentioned in the introduction, a potential confounder of the 17-domain NPCCSS is its complexity and the inherent difficulty of its implementation in everyday clinical practice. The 5-domain abbreviated scale has practical benefits in improving time efficiency, minimizing statistical variability, and reducing influence from disease manifestations that can cause general clinical fluctuations such as seizures and respiratory complications.

NPC disease severity and complications may also fluctuate day to day in individuals with NPC, although this confounder is minimized by the format of the 5-domain NPCCSS, which means that no single domain can be deemed the most important. As a total score, the 5-domain NPCCSS captures the impact of NPC on patients’ functioning, so that each step on the score reflects a considerable change in function. The 5-domain NPCCSS total score differs from tools developed to assess subtle changes in different domains, such as neuropsychological batteries used to assess potential cognitive impairment, the Scale for Assessment and Rating for Ataxia [45], the 9-Hole Peg Test for fine motor changes [46], and the 3-min stair climb test for ambulation. Although these cognitive evaluations have been validated in other disease populations, they are not disease specific and there are not enough data on their specificity and sensitivity regarding rates of change in NPC populations.

Missense mutations in *NPC1* are the most common type of mutation reported in NPC (75% of patient alleles) [12]; this was also the case in this study. As in most lysosomal storage diseases, a clear genotype/phenotype relationship is difficult to establish in NPC [47, 48], and it was not possible to correlate the biomarker and progression values reported here to specific mutations in a meaningful way. One exception was a patient homozygous for the A1108fs frameshift mutation, who exhibited a disease progression of 14 points in 13 months, confirming that double functional null genotypes are highly predictive of a severe and early progressive disease course [12, 47]. Other NPC cohorts of similar sizes have also reported individuals with double functional null genotypes occurring at similar rates within representative, heterogenous populations [49, 50]; therefore, the occurrence of this genotype in the current study is not unexpected. A detailed report on the mutations found in this study population, and in that of the double-blind interventional NPC-002 study, will be explored further in a separate publication.

This study aimed to characterize novel NPC disease biomarkers in order to establish a set of biomarkers for NPC that are focused on interventions targeting the HSP system in NPC and the underlying cellular pathology of NPC, including altered NPC1/2 protein function and lipid metabolism. During the time of this study and its publication, it is worth noting that other biomarkers such as lyso-sphingomyelin-509 and bile acids have also shown potential as biomarkers of NPC disease [41, 51–53].

Determination of unesterified cholesterol in skin biopsy samples showed significantly increased levels in individuals with NPC (*p* = 0.0006) compared with a cohort of healthy individuals whose biomarker levels were representative of the wider population. Hence, the findings confirm that LC–MS/MS-based measurements of skin biopsy samples are a valid method to support the biochemical diagnosis of NPC [21], and to measure the cholesterol storage burden in individuals with NPC. No evidence of changes in the storage burden over time were observed, nor were any correlations with the 5-domain NPCCSS found (Fig. 5a). A limitation of these data was that at Visit 2, skin unesterified cholesterol assessments were performed on fewer individuals than at Visit 1; a minimum of 4 mg skin was required to perform the analysis, and 16 of 36 skin biopsy samples from Visit 2 did not fulfil this requirement. As a result, the repeat assessment for these individuals could not be confirmed. Nevertheless, the levels in the NPC individuals here are similar to those reported previously using similar LC–MS/MS methods [17]. These findings also demonstrate that unesterified cholesterol may be measured directly in skin biopsy samples in lieu of isolating and culturing fibroblasts for subsequent filipin staining, which is both time and labour intensive. This therefore has the potential to shorten the time for diagnosis [21]. To potentially monitor longitudinally unesterified cholesterol levels in individuals with NPC, the PBMC method has benefits over skin biopsy sampling because it eases the sampling burden on the individual, especially within the young NPC demographic, and may reduce variability.

In clinical practice, cholestane-triol is frequently used as a biomarker to support diagnosis [7]. In this trial, cholestane-triol levels are similar to those reported in other NPC cohorts at diagnosis [23, 41]. An increase over 6 months in cholestane-triol levels was observed with a mean (± SD) change per 6 months of 2.64 ng/mL (± 10.69), and cholestane-triol levels were significantly correlated (Spearman’s correlation coefficient = 0.265, *p* = 0.0411) at the population level with disease severity on the 5-domain NPCCSS (Fig. 5c). These results confirm previous studies demonstrating an increased cholestane burden with NPC disease severity [22]. However, unlike previous studies, the CT-ORZY-NPC-001 results reported here do not support a correlation between serum cholestane-triol levels and age of NPC disease onset (data on file) [22, 41]. Together these data support the use of cholestane-triol as a biomarker of disease severity in the NPC population as a whole and may be useful in the follow-up of an individual patient to potentially monitor disease.

Based on the Vanier 1988 method [25], a new analytical method for cholesterol esterification in PBMC was established, aiming to shorten the time to diagnosis and reduce the burden on the individual, while also giving a direct measurement of NPC protein function. The findings demonstrate that cholesterol esterification is significantly decreased in the PBMCs of individuals with NPC compared with healthy individuals (*p* < 0.0001). Mutations in the *NPC* genes either result in reduced protein function because of limited binding capacity, or reduced abundance because of a more rapid degradation of the dysfunctional protein [2, 4, 54, 55]; therefore, NPC function is not expected to change with disease progression. Accordingly, no significant change in cholesterol esterification was observed during the study, nor did cholesterol esterification correlate with disease progression (Fig. 5d). Although variation of cholesterol esterification levels in PBMC was high, both within and between individuals, this was likely owing to the limited viability of PBMCs for the analytical method. Nonetheless, the current study confirms that PBMCs are a valid matrix on which to assess NPC protein function and to potentially support NPC diagnosis.

Establishing the basal levels of HSP70 in the PBMCs of individuals with NPC and assessing its variability during the study was the key intention of measuring HSP70 expression levels. We have established that HSP70 expression levels are decreased in individuals with NPC compared with those in the cohort of healthy individuals, with relatively stable levels over time. These findings are consistent with those demonstrated in murine models where *Npc1*^*−/−*^ mice exhibit lower levels of HSP70 expression and HSF1 activation compared with wildtype mice; drug-induced elevated expression of HSP70 in the *Npc1*^*−/−*^ mouse model was associated with reduction in glycosphingolipid accumulation, improvement in CNS myelination, improvement in measurable manifestations of ataxia and reduced loss of motor coordination [27]. Additionally, insufficient induction of HSPs has been associated with a variety of chronic neurological diseases [26]. The data presented here on HSP70 expression levels in the PBMCs of healthy individuals are in accordance with those previously published in Madden et al. 2010 [56].

With regard to safety, this was a non-interventional study with no investigational medicinal product, and so AEs were recorded to inform the background occurrence of adverse events in individuals with NPC on routine clinical care. Overall, nine of the 120 AEs (7.5%) were considered definitely related to NPC disease and eight AEs (6.7%; all cases of diarrhoea) were considered to be related to miglustat use by the investigators. This highlights the importance of ensuring stable routine clinical care prior to enrolment.

Among the potential limitations of the NPC-001 observational study was the high proportion of individuals receiving miglustat (30/36; 83.3%). Miglustat is likely to modify the clinical progression of NPC [57]. However, the nearly ubiquitous use of miglustat across the study population meant that subgroup analysis of those not receiving miglustat could not be done, and so the effect of miglustat use on clinical evolution and disease progression could not be determined. With regard to the baseline disease characteristics as reported by referring clinicians as current medical conditions, the specific term of ataxia (reported in 2 of 36 individuals [5.6%]) appeared to be lower than in other reported NPC populations. This is likely because many of the study participants were defined non-specifically at baseline as clumsy or uncoordinated, rather than given the specific term of ataxic. However, from the NPC-cdb tool during baseline study assessments, ataxia was found to be present in 28 of 36 individuals (77.8%); this is in line with both the ambulation domain scores of the current study population and with the literature of other reported populations, in which it may be present in up to 76% of individuals with NPC [1, 10, 58]. Ideally, to optimize an assessment of NPC disease progression, the study could have been improved by a longer duration and more frequent or intermediary assessments (e.g. 6-monthly) than over the 6–14-month observation period employed here.

Another study limitation was the lack of standardization of skin punch biopsy depth across the clinical study sites. Although all analyses were performed by a single central laboratory, the biopsy samples were collected by different clinicians at different study sites and some biopsy samples were not large enough to apply the analysis method. Additionally, this variability was likely also influenced by the challenge of acquiring skin biopsy samples from children, the quality of which is dependent on skin thickness and elasticity, which is determined by the precise site of biopsy and age of individual.

The reliability study of the 5-domain NPCCSS, conducted to verify the accuracy of the scoring method between and within raters, provided additional support for the use of the 5-domain NPCCSS. The results support the use of the 5-domain NPCCSS score as a reliable endpoint in future studies and suggest that clinicians were able to rate participants similarly and consistently, as well as utilize each of the categorical severity ratings in the manner intended. The supportive Many-Facets Rasch analysis illustrated that each of the items contributing to the 5-domain NPCCSS were locally independent (i.e. not dependent on responses to other items), and that the instrument, in its brevity, is able to target participants along the severity continuum with precision.

## Conclusions

In this study, the disease course of patients with NPC, a severe progressive neurodegenerative disease, was prospectively characterized over a period of 6–14 months using clinical measures and biomarkers. In line with previous reports, disease progression pattern and rate were heterogeneous in this population. Most patients exhibited disease progression during the observation period as assessed by the 5-domain NPCCSS and by the overall 17-domain NPCCSS. Notably, the abbreviated version showed high correlation with the 17-domain NPCCSS, which had similar progression rates to those previously reported. These findings confirm the 5-domain NPCCSS as a suitable abbreviated outcome measure in NPC that is utilized as the primary outcome measure in the ongoing interventional clinical trial in NPC (CT-ORZY-NPC-002; ClinicalTrials.gov ID: NCT02612129) [59].

In addition, biomarker results indicate that cholestane-triol may be used as a disease monitoring biomarker with the potential to assess biological response to pharmacological treatment. The results from this study further confirmed the relevance of unesterified cholesterol and cholesterol esterification in the biochemical diagnosis of NPC, independent of disease severity. The finding that the levels of HSP70 in individuals with NPC are decreased compared with a cohort of healthy individuals is particularly interesting given the role of HSP70 in ensuring proper NPC1 function [28], and the mechanism of action of arimoclomol. Arimoclomol is an HSP70 amplifier being evaluated for its efficacy and safety in the CT-ORZY-NPC-002 clinical trial in NPC [59]. In that trial, HSP70 is measured to assess the pharmacodynamic effect of arimoclomol treatment on the heat shock protein response.

The inter- and intra-rater reliability analysis represent a key step in the full validation process. The ongoing validation work will be published elsewhere once finalized. However, based on the data and analyses presented here, the 5-domain NPCCSS scale can be considered as a reliable outcome measure in other clinical studies.

## Methods

### Observational study (CT-ORZY-NPC-001)

#### Study design

This was a prospective, multicentre, observational study to establish disease natural history in which individuals remained on their routine clinical care. It was performed at 12 clinical sites in seven countries over an observation period of 6–14 months. Study assessments were performed at screening and enrolment (Visit 1) and up to 40 weeks later at the end of the study (Visit 2). Telephone follow-ups were every 8 weeks. The primary objective was clinical progression assessed by the 5-domain and 17-domain NPCCSS, the NPC-cdb score and a quality-of-life questionnaire; the secondary objective of this study was safety relating to disease progression and routine clinical care. Additional exploratory endpoints included biomarker analyses of HSP70 in PBMCs, cholesterol esterification in PBMCs, serum cholestane-triol, unesterified cholesterol in PBMCs and skin biopsy samples, and genotype analysis. The study adhered to the Declaration of Helsinki, International Conference on Harmonization (ICH) and Good Clinical Practice (GCP) standards, and applicable local guidelines [60, 61]. The study protocol and associated documentation were approved by the relevant independent ethics committees and/or institutional review boards, and written informed consent was obtained at enrolment from either the individual or their legal guardian.

#### Study participants

The inclusion criteria for the study specified that participants should be: male or female; aged 2 to 18 years (to exclude individuals with severe neonatal onset of disease); of any ethnicity; with a genetic diagnosis of either NPC1 or NPC2; with a body mass index (BMI) Z score of ≥ − 2 SD below mean of the age-adjusted population; presenting with at least one neurological symptom; able to walk with assistance; treated or non-treated with miglustat with stable dosing for at least 3 months; and, for those applicable, receiving adequate contraception. Potential participants were excluded if they exhibited: uncontrolled epilepsy; severe hepatic or renal insufficiency; historic or planned liver transplants; and other severe disease manifestations that would inhibit compliance. Potential participants were also excluded if they or their legal guardians did not provide written informed consent.

### Clinical progression

Disease progression was assessed by establishing baseline characteristics at Visit 1 and calculating both the absolute change at Visit 2 and the change in parameter per 6 months (change between visits divided by time between visits times 6 months). The total 17-domain NPCCSS score consists of nine major domain scores of 0–5 and eight minor domain scores of 0–2. Summation of these 17 domains yields total scores that ranged from 0 to 61, with a higher score indicating a more severe clinical impairment.(15) The abbreviated 5-domain NPCCSS consists of five key domains that were determined to be the most clinically relevant to individuals with NPC, caregivers and clinicians: ambulation, cognition, fine motor skills, speech and swallowing. The 5-domain NPCCSS total score ranges from 0 to 25, with a higher score indicating a more severe clinical impairment. The NPC-cdb score aims to reflect an individual’s clinical status. It ranges from 0 to 125 and an increase in score reflects a reduction in the individual’s abilities [62]. In this study, the NPC-cbd score was modified to utilize a simplified scoring system. Health-related quality of life (QoL) was assessed according to the EQ-5D-3L Y questionnaire [63].

#### Genotype analysis

Genotype analyses were sourced from historical participant records and individual alleles were categorized according to mutation type.

#### Biomarkers

### Unesterified cholesterol

Whole blood samples were collected in K2-EDTA tubes and shipped to the central analysis site for isolation of the PBMCs. Pellets of 1 million cells were generated and stored at –80 °C. Lipids were extracted followed by derivatization and unesterified cholesterol was quantified by LC–MS/MS as per Crosley et al*.* 2009 [64]. Skin punch biopsy samples were collected using a 3 mm biopsy plunger (Miltex 33-32P; Integra LifeSciences, Plainsboro Township, NJ, USA) and stored at –80 °C. Lipids were extracted followed by derivatization and unesterified cholesterol was quantified by LC–MS/MS.

### Cholestane-triol

Serum samples were collected in serum separation tubes and immediately separated by centrifugation at each clinical site. Samples were stored at –80 °C and shipped to the central analysis site. Cholestane-triol was determined by LC–MS/MS.

### Cholesterol esterification

Whole blood samples were collected in sodium-heparin tubes and shipped at ambient temperature to the central analysis site. PBMCs were isolated by application of a density gradient and incubated for 48 h with heptadecanoic acid (C17). Following cellular esterification of cholesterol with the heptadecanoic acid, cholesterol heptadecanoate was quantified by LC–MS/MS.

### HSP70

Whole blood samples were collected in K2-EDTA tubes and shipped to the central analysis site for isolation of the PBMCs. Pellets of 1 million cells were generated and stored at –80 °C. Frozen cell pellets were homogenized in RIPA buffer with 1% Protease and Phosphatase Inhibitor Cocktail (R0278, P0044, P8340; Sigma-Aldrich, St. Louis, MO, USA), centrifuged, and extracts were diluted in assay buffer. HSP70 levels were then quantified by sandwich enzyme-linked immunosorbent assay (ELISA) (DYC1663; Research and Diagnostic Systems, Inc., Minneapolis, MN, USA).

All analytical methods were validated and met the acceptance criteria for inter-assay accuracy and precision at 15% for LC–MS/MS methods and 20% for the ELISA assay (sensitive to haemolysis). No interfering peaks were detected at the retention time of the internal standard or the analytes for the chromatographic assays. No carry over was observed.

#### Safety

All AEs and SAEs were recorded, as were any changes in the results of physical examinations, vital signs, electrocardiographic results, and standard haematology and clinical chemistry findings.

#### Statistical analyses

Continuous variables were summarized by descriptive statistics and categorical variables were assessed by absolute and relative frequencies; results from the EQ-5D-3L Y analyses were assessed according to the Pareto principle as per Devlin et al*.* 2010 [65]. For biomarker data, comparisons between visits utilized the Wilcoxon–Mann–Whitney test and comparisons between individuals with NPC and healthy individuals utilized the Wilcoxon signed-rank test; data from healthy individuals were representative of those reported in the wider healthy population. All statistical analyses were performed using SAS v9.3 (SAS Institute, Cary, NC, USA).

### Reliability study of the 5-domain NPCCSS (OR-REL-NPC-01)

#### Study design

Four videos with a clinical evaluation of four different individuals with NPC were recorded and formed the basis of this study. All individuals provided informed consent and parental or guardian permissions to use the videos. A single medical NPC expert performed a medical interview and physical examination of each of the four individuals; the format of each of the videos was standardized. Altogether, individuals were selected to represent a wide range of disease burden. The NPC expert evaluated each individual in accordance with a written 5-domain NPCCSS rater manual, taking into account the individual’s age and level of disability.

The four video-recorded clinical evaluations were then evaluated by a group of 13 clinician raters to assess inter- and intra-rater reliability of the 5-domain NPCCSS. The clinicians received standardized training as per the rater manual and the training provided in the CT-ORZY-NPC-001 observational study.

Raters scored each video twice. An initial scoring was conducted for each of the four individuals on the 5-domain NPCCSS and was then repeated in a random order, with at least a 3-week interval between repeat ratings. The 13 clinicians were blinded to other raters’ scores.

#### Study participants (video recording)

Eligibility for participation was limited to individuals with NPC1 (aged 2–18 years) with at least one neurological symptom, the ability to walk independently or with assistance, and both participant and their caregiver fluent in English. Individuals participating in NPC-001 or NPC-002 clinical trials were ineligible.

#### Statistical analysis

A power calculation was conducted a priori to estimate the number of raters needed to rate the videos in order to derive reliability estimates above the 0.7 acceptable reliability threshold. To allow for a power of approximately 80% to detect within-group consistency and null between-group variability, the targeted sample size was 14 raters. In the study, 13 raters were recruited, each of them provided initial and repeat ratings for each video, thus constituting the analytical data set. Scores provided by each rater on the 5-domain NPCCSS (range 0–25) at the second rating were compared with the first rating in order to assess intra-rater reliability. A two-way analysis of variance (ANOVA) with repeated measures on raters and participants was used to calculate inter- and intra-rater reliability within a single model for the 5-domain NPCCSS. For the individual domains (ambulation, cognition, fine motor skills, speech and swallowing), Kendall’s coefficient of concordance (W) and polychoric correlations were evaluated independently for intra-rater reliability. For each reliability parameter, the intraclass correlation coefficients (ICC2,13) were generated with pre-specified criteria for both defined as 0.70 (range 0–1.0 with a higher threshold reflective of better rater agreement and stability) [66]. As a sensitivity analysis to identify potential error in the reliability estimates due to rater bias, a Many-Facets item-response analysis was conducted, which allows the clinician rating of severity to be derived using a standardized scale similar to the individual and to the analyzed item (i.e. equating to a similar metric) [42].

## Data Availability

All data and materials were available to authors. The data that support the findings of this study are available from Orphazyme but restrictions apply to the availability of these data, which were used under license for the current study, and so are not publicly available. Data are however available from the authors upon reasonable request and with permission of Orphazyme.

## Abbreviations

AE: Adverse event
ANOVA: Analysis of variance
BMI: Body mass index
ELISA: Enzyme-linked immunosorbent assay
EQ-5D-3L Y: EuroQol 5-Dimension 3-Level Youth Proxy version questionnaire
GCP: Good Clinical Practice
HSP: Heat shock protein
HSR: Heat shock response
ICC: Intraclass correlation coefficient
ICH: International Conference on Harmonization
LC–MS/MS: Liquid chromatography-mass spectrometry
MCID: Minimal clinically important difference
NPC: Niemann–Pick disease type C
NPC-cdb: NPC clinical database
NPCCSS: NPC Clinical Severity Scale
NS: Not significant
PBMC: Peripheral blood mononuclear cell
SAE: Serious adverse event
SD: Standard deviation
SEM: Standard error of the mean
