# Correction to: Clinical disease progression and biomarkers in Niemann–Pick disease type C: a prospective cohort study

**DOI:** 10.1186/s13023-021-01855-9

**Published:** 2021-06-01

**Authors:** Eugen Mengel, Bruno Bembi, Mireia del Toro, Federica Deodato, Matthias Gautschi, Stephanie Grunewald, Sabine Grønborg, Bénédicte Héron, Esther M. Maier, Agathe Roubertie, Saikat Santra, Anna Tylki-Szymanska, Simon Day, Tara Symonds, Stacie Hudgens, Marc C. Patterson, Christina Guldberg, Linda Ingemann, Nikolaj H. T. Petersen, Thomas Kirkegaard, Christine í Dali

**Affiliations:** 1 SphinCS GmbH, Institute of Clinical Science for LSD, Hochheim, Germany; 2 Regional Coordinator Centre for Rare Diseases, Academic Hospital Santa Maria Della Misericordia, Udine, Italy; 3 Vall D’Hebron University Hospital, Barcelona, Spain; 4 Ospedale Pediatrico Bambino Gesù, IRCCS, 00146 Rome, Italy; 5 Inselspital, University Hospital of Bern, Bern, Switzerland; 6 Metabolic Department, Great Ormond Street Hospital NHS Foundation Trust, Institute for Child Health, NIHR Biomedical Research Centre UCL, London, UK; 7 Centre for Inherited Metabolic Diseases, Copenhagen University Hospital (Rigshospitalet), Copenhagen, Denmark; 8 Reference Centre for Lysosomal Disease, Trousseau University Hospital, Paris, France; 9 Dr. Von Hauner Children’s Hospital, University of Munich, Munich, Germany; 10 Institute of Neurosciences, University Hospital of Montpellier, Montpellier, France; 11 Birmingham Children’s Hospital, Birmingham, UK; 12 Children’s Memorial Health Institute, Warsaw, Poland; 13 Clinical Trials Consulting and Training Limited, Buckingham, UK; 14 Clinical Outcomes Solutions Limited, Folkestone, UK; 15 Clinical Outcomes Solutions Inc, Tucson, AZ USA; 16 Mayo Clinic Children’s Center, Rochester, MN USA; 17 Orphazyme A/S, Copenhagen, Denmark

## Correction to: Orphanet Journal of Rare Diseases (2020) 15:328 https://doi.org/10.1186/s13023-020-01616-0

Following the publication of the original article [1], it was brought to the authors’ attention that there are several errors within the article, introduced through both typographical and editorial oversight as well as through final typesetting.

The errors and their respective corrections are shown in Table 1. The authors apologise for these errors, but note that the results and conclusions of the manuscript remain unchanged.

**Table 1:** .

**Error**	**Correction**
Abstract background, page 1: cholestane-3*β*,5*α*-,6*β*-triol	cholestane-3β,5α,6β-triol
*Abstract results, page 1, lines 4-7:* Compared with healthy individuals, the NPC population had significantly lower levels of cholesterol esterification (p < 0.0001), HSP70 (p < 0.0001) and skin unesterified cholesterol (p = 0.0006). Cholestane-triol levels were significantly higher in individuals with NPC versus healthy individuals (p = 0.008) and correlated with the 5-domain NPCCSS (Spearman’s correlation coefficient = 0.265, p = 0.0411).	Compared with healthy individuals, the NPC population had significantly lower levels of cholesterol esterification (p < 0.0001) and HSP70 (p < 0.0001), and significantly higher levels of skin unesterified cholesterol (p = 0.0006). Cholestane-triol levels were also significantly higher in individuals with NPC versus healthy individuals (p = 0.008) and were correlated with the 5-domain NPCCSS (Spearman’s correlation coefficient = 0.265, p = 0.0411).
*Abstract trial registration, page 2:* 2014–005,194-37	2014-005193-37
*Background, page 2, paragraph 2, line 9:* that affect gross motor skills, swallowing ability cogni-	that affect gross motor skills, swallowing ability and cogni-
*Results, page 4, paragraph 4, line 11:* tion still had an end-of-trial visit and provided data for	tion still had an end of study visit and provided data for
*Results, page 4, paragraph 6, line 3:* by referring clinicians as current medical conditions, was	by referring clinicians as current medical conditions, were
*Results, page 4, paragraph 7, line 9:* (Fig. 3b).	(Fig. 3c).
*Figure 3 legend, line 4,* an end-of-trial visit and is included in efficacy assessments	an end of study visit and is included in efficacy assessments
*Table 3, row 1:* Total number of different alleles	Total number of distinct alleles
*Table 4, title:* Change in disease biomarkers over the 6–14-month observation period compared with those of healthy individuals	Disease biomarkers over the 6–14-month observation period and those of healthy individuals
*Results, page 8, paragraph 4, line 20:* (Table 6).	(Table 7).
*Figure 5 legend, line 1-2:* **a.** PBMC unesterified cholesterol. **b.** Skin unesterified cholesterol.	**a.** Skin unesterified cholesterol. **b.** PBMC unesterified cholesterol.
*Table 5, line 20:* Serious adverse events [indented]	This is incorrectly indented, please align to the left.
Table 6	Table 7
Table 7	Table 6
*Results, page 11, paragraph 2, line 12:* (Table 7).	(Table 6.)
*Table 7, heading:* MEAN 5-domain NPCCSS score (±SD)	Mean 5-domain NPCCSS score (±SD)
*Discussion, page 12, paragraph 1, line 12-13:* date and to confirm the patients and clinicians view on clinical meaningful changes on the 5-domain NPCCSS	date and to confirm the patients’ and clinicians’ views on clinically meaningful changes on the 5-domain NPCCSS
*Discussion, page 13, paragraph 2, line 12:* tane burden with NPC disease severity [22]. However,	tane triol burden with NPC disease severity [22]. However,
*Discussion, page 13, paragraph 3, line 19:* this was likely owing to the limited viability of PBMCs	this was likely due to the limited viability of PBMCs
*Discussion, page 14, paragraph 3, line 11:* uting to the 5-domain NPCCSS were locally independent	uting to the 5-domain NPCCSS was locally independent
*Methods, page 15, paragraph 2, line 9:* Incorrect formatting of citation (15)	Delete
*Methods, page 16, paragraph 6, line 22:* Incorrect formatting of abbreviation and citation (ICC2,13)	(ICC) [2,13]
*Abbreviations:* MCID: Minimal clinically important difference;	Delete
